# Invasive disease‐free and overall survival after (neo)adjuvant chemotherapy in postmenopausal patients with hormone receptor‐positive, HER2‐negative early breast cancer treated with upfront letrozole: Experiences from the phase IV PreFace trial

**DOI:** 10.1002/ijc.70037

**Published:** 2025-07-11

**Authors:** Milena Beierlein, Lothar Häberle, Naiba Nabieva, Nicolai Maass, Bahriye Aktas, Sherko Kümmel, Christoph Thomssen, Christopher Wolf, Hans‐Christian Kolberg, Cosima Brucker, Wolfgang Janni, Peter Dall, Andreas Schneeweiss, Frederik Marme, Marc W. Sütterlin, Matthias Ruebner, Anna‐Katharin Theuser, Nadine M. Hofmann, Sybille Böhm, Katrin Almstedt, Sara Kellner, Paul Gass, Hans‐Joachim Lück, Alexander Hein, Sabine Schmatloch, Matthias Kalder, Christoph Uleer, Ingolf Juhasz‐Böss, Volker Hanf, Christian Jackisch, Volkmar Müller, Brigitte Rack, Erik Belleville, Diethelm Wallwiener, Achim Rody, Claudia Rauh, Chistian M. Bayer, Sabrina Uhrig, Hanna Huebner, Chloë Goossens, Sara Y. Brucker, Carolin C. Hack, Tanja N. Fehm, Peter A. Fasching

**Affiliations:** ^1^ Department of Gynecology and Obstetrics, Comprehensive Cancer Center Erlangen‐EMN University Hospital Erlangen, Friedrich‐Alexander‐Universität Erlangen‐Nürnberg Erlangen Germany; ^2^ Biostatistics Unit, Erlangen University Hospital, Department of Gynecology and Obstetrics, Comprehensive Cancer Center Erlangen‐EMN Friedrich‐Alexander University Erlangen‐Nuremberg Erlangen Germany; ^3^ Department of Gynecology and Obstetrics University Hospital of Schleswig‐Holstein, Campus Kiel Kiel Germany; ^4^ Department of Gynecology University Hospital Leipzig Leipzig Germany; ^5^ Breast Unit, Kliniken Essen‐Mitte Essen Germany; ^6^ Charité—Universitätsmedizin Berlin, Department of Gynecology with Breast Center Berlin Germany; ^7^ Department of Gynaecology Martin‐Luther‐University Halle‐Wittenberg Halle (Saale) Germany; ^8^ Medical Center Ulm Ulm Germany; ^9^ Department of Gynecology and Obstetrics Marienhospital Bottrop Bottrop Germany; ^10^ Department of Gynecology and Obstetrics, University Hospital Paracelsus Medical University Nuremberg Germany; ^11^ Department of Gynecology and Obstetrics Ulm University Hospital Ulm Germany; ^12^ Department of Gynecology Lüneburg Clinic Lüneburg Germany; ^13^ Division of Gynecologic Oncology, National Center for Tumor Diseases University Hospital and German Cancer Research Center Heidelberg Germany; ^14^ Department of Gynecology and Obstetrics, University Medical Centre Mannheim, Medical Faculty Mannheim Heidelberg University Mannheim Germany; ^15^ Institut für Frauengesundheit GmbH Erlangen Germany; ^16^ Department of Obstetrics and Gynecology University Medical Center Mainz, Johannes Gutenberg University Mainz Germany; ^17^ Klinikum Chemnitz gGmbH Medizincampus Chemnitz der Technischen Universität Dresden Dresden Germany; ^18^ Gynäkologisch‐Onkologische Praxis Hannover Hannover Germany; ^19^ Department of Gynecology and Obstetrics Klinikum Esslingen Esslingen Germany; ^20^ Elisabeth Krankenhaus Kassel Kassel Germany; ^21^ Department of Gynecology and Obstetrics University Hospital Gießen and Marburg Marburg Germany; ^22^ Gyn.‐onkologische Gemeinschaftspraxis Hildesheim Hildesheim Germany; ^23^ Department of Obstetrics and Gynecology Freiburg University Hospital Freiburg Germany; ^24^ Frauenklinik, Klinikum Fürth Fürth Germany; ^25^ Frauenklinik Sana Klinikum Offenbach Germany; ^26^ Department of Gynecology Hamburg‐Eppendorf University Medical Center Hamburg Germany; ^27^ ClinSol GmbH & Co. KG Würzburg Germany; ^28^ Department of Obstetrics and Gynecology University of Tuebingen Tuebingen Germany; ^29^ Department of Gynecology and Obstetrics University Hospital Schleswig‐Holsteinm, Campus Lübeck Lübeck Germany; ^30^ Department of Gynecology University Hospital Inselspital Bern Bern Switzerland; ^31^ Kreisklinik Ebersberg Ebersberg Germany; ^32^ Department of Gynecology and Obstetrics University Hospital Düsseldorf Düsseldorf Germany; ^33^ Centrum für Integrierte Onkologie Aachen Bonn Köln Düsseldorf Düsseldorf Germany

**Keywords:** adjuvant chemotherapy, breast cancer, hormone receptor‐positive/HER2‐negative, neoadjuvant chemotherapy, phase IV clinical trial

## Abstract

Patients with hormone receptor‐positive (HRpos), HER2‐negative (HER2neg) breast cancer (BC) benefit less from neoadjuvant chemotherapy (NACT) than patients with triple‐negative and HER2‐positive BC. In this retrospective analysis of the phase IV PreFace clinical trial (NCT01908556), where postmenopausal HRpos BC patients (*n* = 3297) were treated with 5‐year upfront adjuvant letrozole therapy, we evaluated the prognosis of patients treated with adjuvant versus neoadjuvant chemotherapy in HRpos/HER2neg early‐stage BC. HRpos/HER2neg patients with information on (neo)adjuvant chemotherapy (*n* = 2895) were retrospectively selected from all patients enrolled in the PreFace trial. Invasive disease‐free survival (iDFS) and overall survival (OS) were compared between patient groups that were treated with neoadjuvant or adjuvant chemotherapy. Chemotherapy was given to 1051 patients (36.3% of all patients), of which 874 (83.2%) received adjuvant chemotherapy and 177 (16.8%) NACT. Pathologic complete response (pCR) rate in the NACT group was 6.9%. Patients treated with NACT had a worse outcome than those treated with adjuvant chemotherapy (5‐year iDFS rate 81% vs. 88%; 5‐year OS rate 89% vs. 93%). This effect was maintained after adjusting for age, BMI, lymph node status, grading, tumor size, and histology (hazard ratio for iDFS: 1.95 (95%CI: 1.28–2.95); hazard ratio for OS: 2.13 (95%CI: 1.24–3.66)). Further adjustment for taxane‐based regimes did not alter results. In conclusion, in this retrospective analysis of patients with early‐stage HRpos/HER2neg BC, patients with NACT had a more unfavorable prognosis than patients treated adjuvantly, independent of patient and tumor characteristics. Prognosis of neoadjuvant patients might be affected by resistance mechanisms, warranting further investigation.

## What's New?

Neoadjuvant chemotherapy (NACT) can be highly effective for patients with early‐stage breast cancer. Patients with hormone receptor‐positive (HRpos), HER2‐negative (HER2neg) breast cancer, however, may benefit less from NACT. Here, the authors investigated survival outcomes with adjuvant chemotherapy or NACT in a population of postmenopausal HRpos/HER2‐neg patients treated with letrozole‐based adjuvant therapy. Analyses show that invasive disease‐free and overall survival rates were higher for patients who received adjuvant chemotherapy versus NACT. Similar results were observed after adjustment for confounders and for taxane‐based regimens. The findings warrant further investigation of drug resistance and prognosis in different populations of early‐stage breast cancer patients treated with NACT.

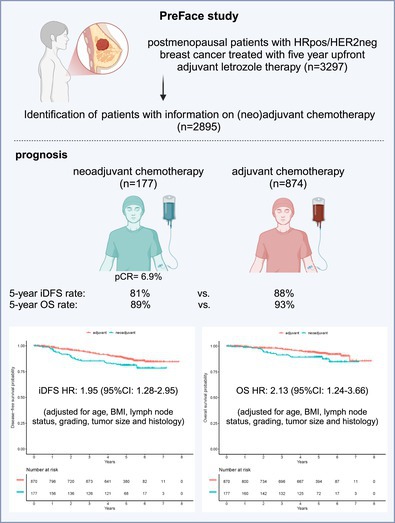

**Abbreviations**
BC: breast cancer
CI: confidence interval
CPS: combined positive score
eBC: early breast cancer
ER: estrogen receptor
FDA: Food and Drug Administration
HER2: humane epidermal growth factor receptor 2
HER2neg: HER2‐negative
HRpos: hormone receptor‐positive
iDFS: invasive disease‐free survival
IQR: interquartile range
NACT: neoadjuvant chemotherapy
OS: overall survival
pCR: pathological complete response
PD‐L1: programmed death ligand 1
PgR: progesterone receptor
TMEM: tumor microenvironment of metastases
TNBC: triple negative breast cancer

## 1 INTRODUCTION

The introduction of neoadjuvant chemotherapy for patients with early‐stage breast cancer (eBC) has changed both clinical practice and the scientific approach to developing novel therapeutics for this patient population. Neoadjuvant treatment can be employed as a first opportunity to test tumor therapy response in vivo, as well as a measure for treatment (de‐)escalation according to the response to said neoadjuvant treatment. Numerous studies have shown that patients who achieve pathologic complete response (pCR) after neoadjuvant treatment have a better prognosis than those not achieving pCR.^1^, ^2^, ^3^, ^4^, ^5^ This is particularly true for patients with triple negative (TNBC) and HER2‐positive (HER2pos) BC.^1^ Importantly, the proportion of patients achieving pCR after neoadjuvant chemotherapy differs between molecular subtypes, with the lowest pCR rates (2%–11%) reported in hormone receptor‐positive (HRpos), HER2‐negative (HER2neg) BC.^6^ Currently, the pCR rate is considered a biomarker for additional postneoadjuvant therapy. After pCR achievement, postneoadjuvant therapy is generally not advised, even though standard adjuvant treatment can be initiated irrespective of pCR. In contrast, patients who do not achieve pCR, thus having a more unfavorable prognosis, can receive postneoadjuvant treatment.^7^

Even though benefits with neoadjuvant therapy have been reported, discussions on whether improved pCR directly translates to better survival are ongoing. Whereas pCR improvement formed the basis for accelerated approval of pertuzumab in the neoadjuvant setting by the Food and Drug Administration (FDA),^8^, ^9^ this approach has not been widely adopted after further scientific investigation into the translation of improved pCR into better survival. Instead, each case is now considered individually.^8^ Additionally, a meta‐analysis indicated that pCR improvement should not be used as a surrogate for prognosis improvement in neoadjuvant clinical trials, not even in TNBC and HER2pos patient populations.^10^

The disconnect between pCR achievement and prognosis in some patients may be due to resistance to neoadjuvant treatment, as well as other factors influencing prognosis after neoadjuvant chemotherapy. Recent data suggest that in certain patients, the residual tumor might change molecular and cellular status and develop an increased likelihood to metastasize.^11^, ^12^, ^13^, ^14^ Furthermore, in HRpos/HER2neg breast cancer, resistance to endocrine treatments can also affect prognosis.^15^ Due to these resistance mechanisms, some patient populations might benefit more from adjuvant than neoadjuvant chemotherapy. In a meta‐analysis that pooled data of 4756 BC patients who received either neoadjuvant or adjuvant chemotherapy between 1983 and 2002, no difference in the risk for distant recurrences or all‐cause mortality was observed.^15^ Nevertheless, there may still be differences according to some patient and tumor characteristics. Due to the large number of patients recruited in the 1980s and 1990s, the hormone receptor status of the majority of patients (74%) in the meta‐analysis was unknown. Furthermore, only one study in this analysis used a taxane‐based chemotherapy regimen, which is now considered the standard.^16^ The limited power of this subgroup analysis underscores the need for additional evidence regarding cancer subtype‐specific resistance to neoadjuvant chemotherapy. Given the high pCR rates observed in patients with TNBC and HER2pos disease, which correlate with a more favorable prognosis, it may be advisable to further evaluate the HRpo/HER2neg population, where pCR rates are relatively low and the discussion into potential therapy de‐escalation strategies is ongoing. Therefore, we here performed a retrospective analysis in a population of postmenopausal patients with early‐stage HRpos/HER2neg BC who had participated in the Phase IV PreFace trial and received a uniform endocrine treatment (5 years of adjuvant letrozole) in order to investigate the effect of neoadjuvant versus adjuvant chemotherapy on prognosis.

## 2 METHODS

### 2.1 Clinical trial

The PreFace Study (Evaluation of PREdictive FACtors Regarding the Effectivity of Aromatase Inhibitor Therapy, NCT01908556) was a prospective, open‐label phase IV clinical trial in patients with HRpos eBC.^17^ The study was conducted between 2009 and 2016 in 220 study sites across Germany. Postmenopausal patients with HRpos eBC were eligible if their attending physician recommended adjuvant upfront letrozole treatment for a duration of 5 years according to the summary of product characteristics for letrozole. No specific requirements regarding risk profiles were made. Letrozole treatment was recommended to begin as soon as possible after final surgery or completion of (neo)adjuvant chemotherapy. The primary analysis has been published elsewhere.^17^

### 2.2 Patients

**FIGURE 1:**
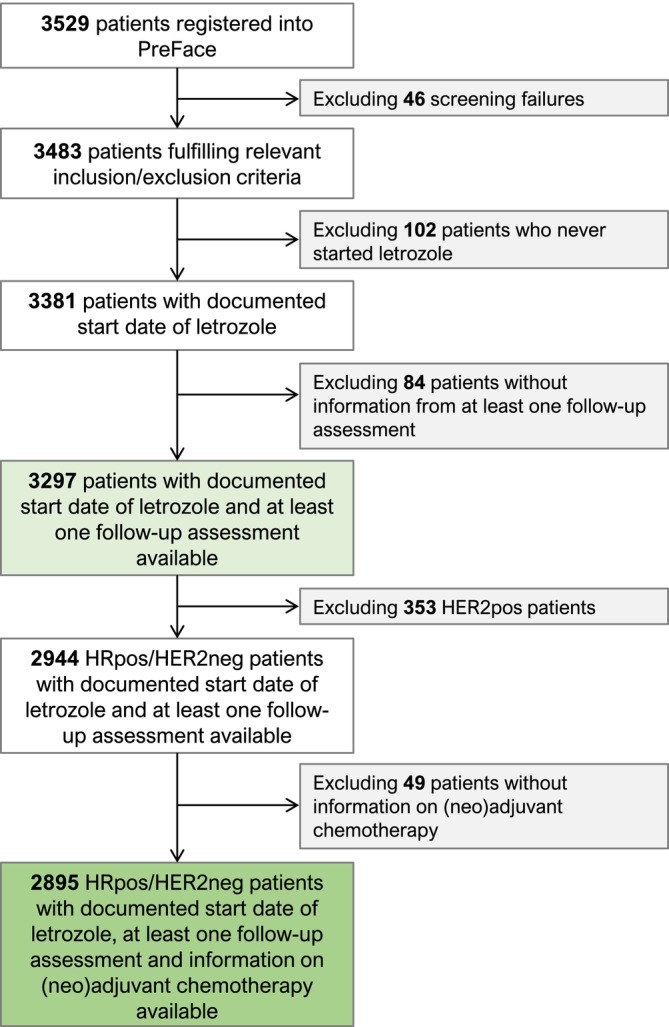
Patient flow chart (CONSORT Diagram). (HRpos, Hormone receptor‐positive; HER2pos, HER2‐positive; HER2neg, HER2‐negative).

HRpos/HER2neg patients with information on neoadjuvant and adjuvant chemotherapy (*n* = 2895) were selected from all patients enrolled in the PreFace study. A study flow chart is presented in Figure 1. HER2pos patients, who were generally eligible for inclusion in the PreFace study, were excluded from this analysis on the basis that HER2pos neoadjuvant treatment has markedly different pCR rates and a relatively high degree of correlation between pCR results and prognosis.^1^ Documentation of (neo)adjuvant chemotherapy and final surgery was part of the PreFace study.

### 2.3 Histopathology

Both hormone receptor and HER2 assessment were recommended in accordance with the ASCO/CAP guidelines.^18^, ^19^ A central review of histopathological assessment or immunohistochemistry was not conducted. In line with guidelines, the estrogen receptor (ER) and progesterone receptor (PgR) status were defined as positive if ≥1% of the cells were stained. A positive HER2 status was defined as an immunohistochemistry score of 3+ or a positive fluorescence in situ hybridization/chromogenic in situ hybridization. Retrospective adjustments for hormone receptor status and HER2 status based on more recent versions of the ASCO/CAP guidelines were not made.

### 2.4 Endpoints

The primary study endpoint was invasive disease‐free survival (iDFS), which was defined from the date of therapy start to the earliest date to relapse (invasive local, regional, and distant recurrences; contralateral breast cancer; second non‐breast primary cancer; and death from any cause) or the last date known to be disease‐free. Overall survival (OS) was a secondary endpoint and defined from the date of therapy begin to the date of death or the last date known to be alive. iDFS and OS were each left‐truncated for time to enter the study, if the entry was after therapy begin. The achievement of pCR was defined as having either ypT0 or ypTis and ypN0.

### 2.5 Statistical methods

Continuous patient and tumor characteristics were summarized as means and standard deviations, and ordinal and categorical characteristics were summarized as frequencies and percentages.

Patients receiving neoadjuvant chemotherapy were compared with those receiving adjuvant chemotherapy in terms of iDFS and OS. Survival rates with 95% confidence intervals (CIs) were estimated using the Kaplan–Meier product limit method. Unadjusted hazard ratios were estimated using a simple Cox regression model; adjusted hazard ratios were estimated using a multiple Cox regression model with age, body mass index, lymph node status, grading, tumor stage, and histology as adjusting variables.

As sensitivity analysis, survival analyses were repeated for patients who were treated with a taxane‐based regimen. For reference purposes, survival rates for HRpos/HER2neg eBC patients from the PreFace trial who were not treated with (neo)adjuvant chemotherapy were also calculated.

Calculations were carried out using the R system for statistical computing (version 4.2.1; R Development Core Team, Vienna, Austria, 2022).

## 3 RESULTS

### 3.1 Patients

The majority of HRpos/HER2neg patients did not receive a neoadjuvant or an adjuvant chemotherapy (*n* = 1844; 63.7% of the complete patient population). Of the patients who were treated with chemotherapy (*n* = 1051), 874 (83.2%) received adjuvant chemotherapy and 177 (16.8%) received neoadjuvant chemotherapy. Patients treated with neoadjuvant chemotherapy were slightly younger compared to patients treated with adjuvant chemotherapy (59.9 ± 7.1 years old vs. 62.0 ± 7.0 years old). Furthermore, the proportion of patients with a positive nodal status at the time of surgery seemed higher in patients who were treated with adjuvant chemotherapy (62.3%) than in the patients who were treated in the neoadjuvant setting (53.4%). Other patient and tumor characteristics were similar across subgroups based on chemotherapy timing (Table 1).

**TABLE 1:** Patient characteristics relative to type of chemotherapy, showing mean and standard deviation (SD) or frequency and percent.

Characteristic		Chemotherapy		
		Neoadjuvant (*N* = 177)	Adjuvant (*N* = 874)	Naïve (*N* = 1844)
Age at study entry (years)	Mean (SD)	59.9 (7.1)	62.0 (7.0)	65.3 (7.6)
	Median (first, third quartile)	58.7 (54.9, 65.7)	61.9 (56.4, 67.3)	65.4 (59.8, 70.3)
	< 65	128 (72.3)	566 (64.9)	885 (48.1)
	≥ 65	49 (27.7)	306 (35.1)	955 (51.9)
BMI (kg/m^2^)	Mean (SD)	27.1 (5.1)	27.4 (5.4)	27.1 (5.0)
	Median (first, third quartile)	26 (23.3, 29.7)	26.4 (23.7, 29.8)	26.3 (23.6, 29.8)
	< 20	8 (4.5)	33 (3.8)	73 (4.0)
	20–25	60 (34.1)	270 (31.3)	629 (34.4)
	25–30	68 (38.6)	351 (40.7)	679 (37.1)
	≥ 30	40 (22.7)	208 (24.1)	450 (24.6)
Lymph node status—*N* (%)	pN0	82 (46.6)	328 (37.7)	1632 (89.3)
	pN+	94 (53.4)	541 (62.3)	195 (10.7)
Tumor size—*N* (%)	pT0/is	16 (9.3)	1 (0.1)	6 (0.3)
	pT1	72 (41.9)	370 (42.4)	1411 (76.6)
	pT2	65 (37.8)	414 (47.4)	386 (21.0)
	pT3	16 (9.3)	72 (8.2)	23 (1.2)
	pT4	3 (1.7)	16 (1.8)	16 (0.9)
Grading—*N* (%)	G1	16 (9.1)	61 (7.0)	494 (26.8)
	G2	110 (62.5)	572 (65.5)	1205 (65.5)
	G3	50 (28.4)	240 (27.5)	141 (7.7)
Estrogen receptor (ER) status—*N* (%)	ER−	5 (2.8)	20 (2.3)	9 (0.5)
	ER+	172 (97.2)	852 (97.7)	1831 (99.5)
Progesteron receptor (PgR) status—*N* (%)	PgR−	29 (16.4)	123 (14.1)	205 (11.1)
	PgR+	148 (83.6)	750 (85.9)	1636 (88.9)
Hormone receptor (HR) status—*N* (%)	ER−/PgR−	1 (0.6)	1 (0.1)	3 (0.2)
	ER−/PgR+	4 (2.3)	19 (2.2)	6 (0.3)
	ER+/PgR−	28 (15.8)	121 (13.9)	202 (11.0)
	ER+/PgR+	144 (81.4)	731 (83.8)	1628 (88.5)
Histology—*N* (%)	Ductal	132 (75.0)	633 (72.6)	1325 (72.0)
	Lobular	38 (21.6)	168 (19.3)	322 (17.5)
	Other	6 (3.4)	71 (8.1)	194 (10.5)

Abbreviations: BMI, body mass index; SD standard deviation.

In the population of patients treated with neoadjuvant chemotherapy, 160 patients (92.0%) received a taxane‐based treatment, whereas in the adjuvant setting, this number was 593 (68.2% of patients). Among patients treated with neoadjuvant chemotherapy, 12 patients (6.9%) achieved pCR.

### 3.2 Survival

**FIGURE 2:**
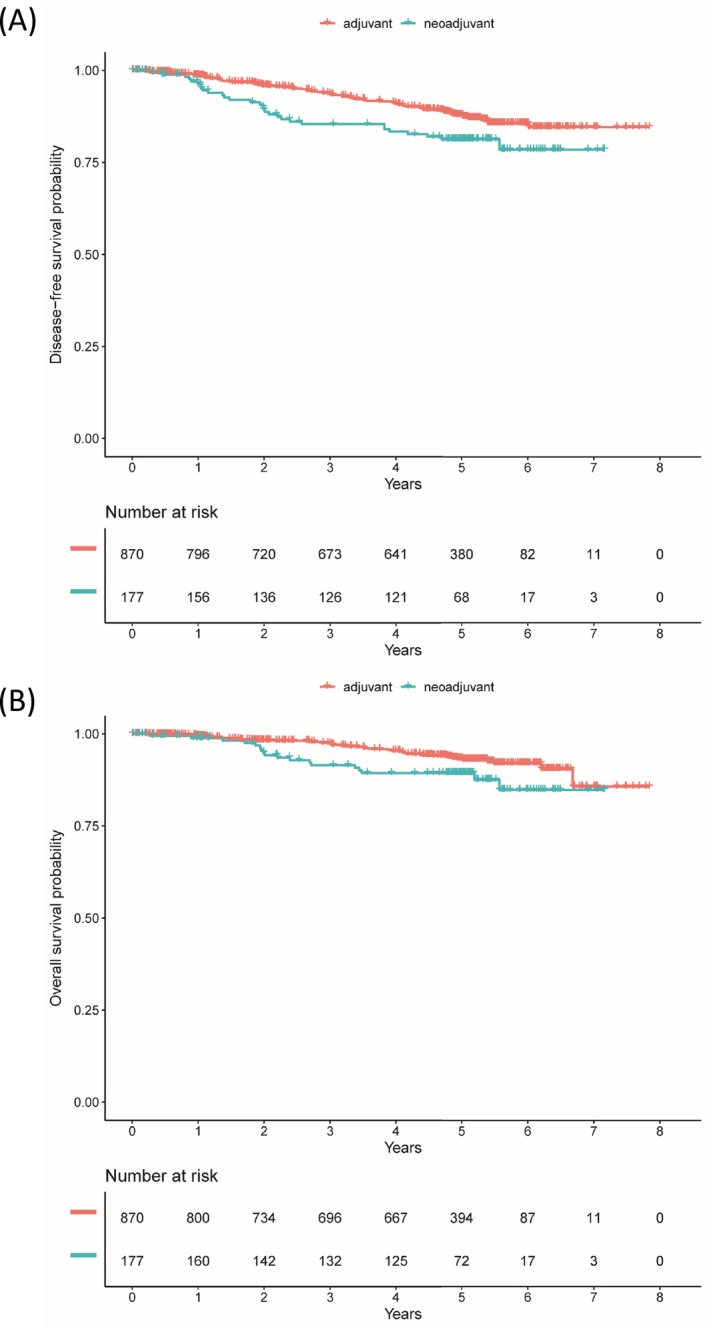
Survival after adjuvant or neoadjuvant chemotherapy. (A) Invasive disease‐free survival. (B) Overall survival.

Median follow up for iDFS was 59.5 (interquartile range [IQR]: 38.9, 51.0) months and 59.5 (IQR: 49.1, 51.3) months for OS. During that observation time, 126 iDFS events and 71 OS events occurred among the 1051 patients treated with chemotherapy. Survival rates for iDFS and OS in the cohorts of patients treated with either neoadjuvant or adjuvant chemotherapy are shown in Table 2 and the respective Kaplan Meier Curves are presented in Figure 2A, B. Patients who were treated with a neoadjuvant chemotherapy had a less favorable iDFS and OS compared to patients who were treated with adjuvant chemotherapy. Five‐year iDFS rate after neoadjuvant chemotherapy was 81% (95% CI: 7%–88%), whereas after adjuvant chemotherapy, the 5‐year iDFS rate was 88% (95% CI: 85–90%). Five‐year OS rates were 89% (95% CI: 85–94%) after neoadjuvant chemotherapy and 93% (95% CI: 91–95%) after adjuvant chemotherapy (Table 2). Considering patient and tumor characteristics, patients who received a neoadjuvant chemotherapy had an increased risk for recurrence compared to patients who received adjuvant chemotherapy (adjusted hazard ratio for iDFS: 1.95; 95% CI: 1.28–2.95). The adjusted hazard ratio for OS was 2.13 (95% CI: 1.24–3.66, Table 3).

**TABLE 2:** Invasive disease‐free survival (iDFS) and overall survival (OS) rates.

Target	Prior chemotherapy	Patients	Events	2‐year survival rate (95% CI)	3‐year survival rate (95% CI)	5‐year survival rate (95% CI)
iDFS	Adjuvant	874	96	0.96 (0.94, 0.97)	0.94 (0.92, 0.95)	0.88 (0.85, 0.90)
iDFS	Neoadjuvant	177	30	0.89 (0.85, 0.94)	0.85 (0.80, 0.91)	0.81 (0.75, 0.88)
OS	Adjuvant	874	53	0.98 (0.97, 0.99)	0.97 (0.96, 0.99)	0.93 (0.91, 0.95)
OS	Neoadjuvant	177	18	0.95 (0.92, 0.98)	0.91 (0.87, 0.96)	0.89 (0.85, 0.94)

Abbreviation: CI, confidence interval.

**TABLE 3:** Hazard ratios for invasive disease‐free survival (iDFS) and overall survival (OS) comparing patients with adjuvant chemotherapy and patients with neoadjuvant chemotherapy.

Target	Prior chemotherapy	Hazard ratio adjusted^a^ (95% CI)	Hazard ratio unadjusted (95% CI)
iDFS	Adjuvant	Reference	Reference
iDFS	Neoadjuvant	1.95 (1.28, 2.95)	1.63 (1.08, 2.45)
OS	Adjuvant	Reference	Reference
OS	Neoadjuvant	2.13 (1.24, 3.66)	1.75 (1.03, 2.99)

Abbreviations: CI, confidence interval; HI, hazard ratio.

^a^ Hazard ratio is adjusted for age, body mass index, lymph node status, grading, tumor size, and histology.

In patients who were treated with a taxane‐based regime, 5‐year iDFS and OS rates remained similar to those in the overall population (iDFS neoadjuvant: 81%; 95% CI: 75%–88%), adjuvant: 87% (95% CI: 84%–90%); OS neoadjuvant: 90% (95% CI: 85%–95%), adjuvant: 92% (95% CI: 90%–94%; Supplementary Table 1). Correspondingly, adjusted hazard ratios for iDFS and OS in patients treated with taxane‐based regimes were also comparable to those of the total population (iDFS: adjusted hazard ratio: 1.81; 95% CI: 1.15–2.86), OS adjusted hazard ratio: 1.61 (95% CI: 0.88–2.93; Supplementary Table 2). The respective Kaplan–Meier curves are presented in Supplementary Figure 1.

Survival rates for patients who were not treated with (neo)adjuvant chemotherapy are shown in Supplementary Table 3. The respective Kaplan Meier curves are shown in Supplementary Figure 2A, B. The more favorable prognostic profile of the group of patients who were not treated with chemotherapy (Table 1) resulted in a group with the numerically best prognosis.

## 4 DISCUSSION

In this exploratory and retrospective analysis of the HRpos/HER2neg patient population of the PreFace trial, we present hypothesis‐generating data that suggest that there may be patient groups that do not benefit as much from neoadjuvant chemotherapy as from adjuvant chemotherapy. In our population of postmenopausal HRpos/HER2neg patients with eBC treated with adjuvant letrozole, iDFS and OS were more favorable in patients who received adjuvant chemotherapy than in those who received neoadjuvant chemotherapy.

Over the past two decades, neoadjuvant chemotherapy contributed to our understanding of how a tumor reacts to chemotherapy in situ. The achievement of pCR after neoadjuvant chemotherapy is associated with a more favorable prognosis,^1^, ^2^ especially in patients with TNBC and HER2pos disease.^1^, ^2^, ^4^, ^5^, ^20^ In patients with HRpos/HER2neg BC, pCR rates after neoadjuvant chemotherapy are lowest, ranging between 2% and11.5%,^6^ which is comparable to the pCR rate of 6.8% reported in this study. Given these low pCR rates in HRpos/HER2neg patients, the indication for neoadjuvant chemotherapy is less clear in this population than it is for TNBC and HER2pos patients. Indeed, while neoadjuvant therapy is the recommended standard for the majority of TNBC and HER2pos patients, guidelines suggest that patients with HRpos/HER2neg disease may be treated with neoadjuvant chemotherapy if there is sufficient patient and disease‐related information that warrants this approach.^21^ Notably, specifically for the HRpos/HER2neg BC patient population, for whom pCR might not be the optimal predictor of outcome, the CPS + EG score (including pre‐treatment clinical stage, post‐treatment pathological stage, ER status and tumor grade) has been suggested as a valuable alternative. This score has been shown to be able to stratify HRpos/HER2neg patients according to outcome (iDFS, OS and locoregional recurrence)^22^, ^23^ and could provide better prognostic information than pCR.^24^ Recent data from the KEYNOTE‐756 study, a randomized controlled trial in which HRpos/HER2neg patients received neoadjuvant chemotherapy with or without pembrolizumab, followed by adjuvant pembrolizumab or placebo in combination with endocrine therapy, reveals a high level of variability in the response to neoadjuvant chemotherapy in patients with HRpos tumors. Subdividing patients based on ER status and programmed death ligand 1 (PD‐L1) status resulted in three patient groups (PD‐L1 combined positive score [CPS]<1 + ER≥10%; PD‐L1 CPS≥1 + ER≥10%; PD‐L1 CPS≥1 + ER <10%) with distinctly different pCR achievement rates. The pCR rates were 2.7% in the PD‐L1 CPS <1 + ER≥10% group, 18.4% in the PD‐L1 CPS≥1 + ER≥10% group, and 33.3% in the PD‐L1 CPS≥1 + ER <10% group.^25^ These findings suggest that pCR achievement may be mediated by specific underlying molecular mechanisms in different patient subgroups, which may in turn be associated with resistance to neoadjuvant chemotherapy. Since PD‐L1 status was not assessed in the PreFace study, we could not evaluate whether PD‐L1 status affected outcome in our patient population.

Another factor that should be considered when discussing neoadjuvant vs. adjuvant chemotherapy is the “tumor microenvironment of metastases” (TMEM), micro‐anatomical structures composed of tumor cells, macrophages and endothelium that allow for cancer cell invasion and dissemination to distant sites.^26^ In a small cohort of 20 patients with HRpos/HER2neg eBC who were treated with paclitaxel followed by doxorubicin and cyclophosphamide, a higher number of TMEMs following neoadjuvant chemotherapy than before therapy begin were observed.^13^ Interestingly, the authors had previously demonstrated that tumors with more TMEM formations are associated with a poorer prognosis and a greater risk of metastasis.^26^, ^27^ Consequently, patients treated with neoadjuvant chemotherapy may be at elevated risk of developing metastases despite a reduction in tumor size.^13^ TMEM formations are detected with immunohistochemical stainings in the tissue. As longitudinal tumor sampling was not performed in the PreFace study, TMEM formations were not analyzed as part of this study. Nevertheless, the data on TMEMs is interesting and potentially useful in assessing the effect of chemotherapy on a tumor, despite its clinical utility requiring further critical discussion.^28^, ^29^

A large meta‐analysis of 10 randomized trials (combined *n* = 4756 patients) that compared neoadjuvant and adjuvant applications of the same chemotherapy concluded that patients treated with either neoadjuvant or adjuvant chemotherapy had a comparable iDFS and OS.^15^ Notably, this dataset only contained one study with anthracyclines and taxanes. Taxanes may be of particular interest, as studies in animal models have shown that paclitaxel caused more TMEM structures.^13^ Unfortunately, evidence in patients remains scarce. The ECTO trial that compared the neoadjuvant and adjuvant treatment of doxorubicin/cyclophosphamide followed by cyclophosphamide/methotrexate/fluorouracil could not demonstrate a difference in relapse‐free survival or OS between groups.^16^ Despite all this, it has to be underlined that the role of taxanes in the treatment of eBC remains uncontested as the addition of taxanes to the treatment regimens has reduced mortality.^30^ The ECTO trial included patients irrespective of molecular subtypes, and a subgroup analysis was never reported. Therefore, this trial may not be very informative of the effects of neoadjuvant, anthracycline/taxane‐based chemotherapies in patients with HRpos/HER2neg eBC, in whom the benefit of a neoadjuvant chemotherapy is the lowest and resistance mechanisms might be most prominent. A recent population‐based cohort study comparing neoadjuvant and adjuvant chemotherapy in a propensity score matched population also did not observe differences in outcome parameters distant disease‐free survival, breast cancer specific survival, and OS between groups.^31^ In this population, 71.1% of neoadjuvant chemotherapy patients received taxanes‐based chemotherapy, while only 51.6% of adjuvant chemotherapy was taxane‐based.^31^ Although our study is a retrospective analysis, it contributes to the existing body of evidence in the discussed context.

This is a retrospective analysis of a study conducted to investigate the effects of upfront adjuvant letrozole therapy. The decision regarding neoadjuvant versus adjuvant chemotherapy was completely at the discretion of the treating physician and was made before enrollment in the PreFace study. As such, the results of this retrospective analysis should be considered hypothesis‐generating, and several sources of potential bias have to be acknowledged. First, the total number of patients receiving (neo)adjuvant chemotherapy is limited, and the number of patients receiving neoadjuvant or adjuvant chemotherapy differs considerably. Furthermore, distinct patient and tumor characteristics could have affected the choice of neoadjuvant vs. adjuvant chemotherapy and the outcome. In general, patient and tumor characteristics were well‐balanced, although more node‐positive cases were present in the patient population that received adjuvant chemotherapy. Although the effect of neoadjuvant chemotherapy was maintained after adding tumor and patient characteristics to a multivariate Cox model, it cannot be guaranteed that all influencing factors were considered. Moreover, the choice of chemotherapy was different between the neoadjuvant and adjuvant setting, with more patients treated with neoadjuvant taxanes. However, adding taxanes to the multivariate model in the sensitivity analysis did not alter outcome. As taxane‐based treatments could be considered the more effective regimen,^30^ it is unlikely that this higher percentage of taxane‐based treatments in the neoadjuvant group could have negatively impacted prognosis.^30^

In summary, this study shows an unfavorable prognosis in patients with HRpos/HER2neg eBC treated with neoadjuvant chemotherapy compared to those treated with adjuvant, mostly anthracycline‐ and taxane‐based chemotherapy. Due to the study design, it is appropriate to consider the data as hypothesis‐generating, leading to the recommendation of further investigation into the mechanism of recurrence. Furthermore, therapy efficacy in relation to therapy resistance should be considered, as prognosis may be affected by both the therapy and the resistance mechanisms themselves.

## AUTHOR CONTRIBUTIONS

**Milena Beierlein:** Conceptualization; investigation; writing – review and editing. **Lothar Häberle:** Writing – review and editing; formal analysis; methodology; writing – original draft; visualization. **Naiba Nabieva:** Investigation; writing – review and editing. **Nicolai Maass:** Investigation; writing – review and editing. **Bahriye Aktas:** Writing – review and editing; investigation. **Sherko Kümmel:** Writing – review and editing; investigation. **Christoph Thomssen:** Writing – review and editing; investigation. **Christopher Wolf:** Writing – review and editing; investigation. **Hans‐Christian Kolberg:** Writing – review and editing; investigation. **Cosima Brucker:** Writing – review and editing; investigation. **Wolfgang Janni:** Writing – review and editing; investigation. **Peter Dall:** Writing – review and editing; investigation. **Andreas Schneeweiss:** Writing – review and editing; investigation. **Frederik Marme:** Writing – review and editing; investigation. **Marc W. Sütterlin:** Writing – review and editing; investigation. **Matthias Ruebner:** Writing – review and editing; investigation. **Anna‐Katharin Theuser:** Writing – review and editing; project administration. **Nadine M. Hofmann:** Writing – review and editing; project administration. **Sybille Böhm:** Writing – review and editing; project administration. **Katrin Almstedt:** Writing – review and editing; investigation. **Sara Kellner:** Writing – review and editing; investigation. **Paul Gass:** Writing – review and editing; investigation. **Hans‐Joachim Lück:** Investigation; writing – review and editing. **Alexander Hein:** Writing – review and editing; investigation. **Sabine Schmatloch:** Writing – review and editing; investigation. **Matthias Kalder:** Writing – review and editing; investigation. **Christoph Uleer:** Writing – review and editing; investigation. **Ingolf Juhasz‐Böss:** Writing – review and editing; investigation. **Volker Hanf:** Writing – review and editing; investigation. **Christian Jackisch:** Writing – review and editing; investigation. **Volkmar Müller:** Writing – review and editing; investigation. **Brigitte Rack:** Writing – review and editing; investigation. **Erik Belleville:** Writing – review and editing; project administration. **Diethelm Wallwiener:** Writing – review and editing; investigation. **Achim Rody:** Writing – review and editing; investigation. **Claudia Rauh:** Writing – review and editing; investigation. **Chistian M. Bayer:** Writing – review and editing; investigation. **Sabrina Uhrig:** Writing – review and editing; data curation. **Hanna Huebner:** Investigation; writing – review and editing. **Chloë Goossens:** Investigation; writing – review and editing; writing – original draft; visualization. **Sara Y. Brucker:** Writing – review and editing; investigation. **Carolin C. Hack:** Writing – review and editing; investigation; conceptualization. **Tanja N. Fehm:** Writing – review and editing; investigation. **Peter A. Fasching:** Conceptualization; methodology; investigation; writing – original draft; writing – review and editing; data curation; funding acquisition; visualization.

## FUNDING INFORMATION

The clinical trial was in part funded by Novartis Deutschland GmbH. The company had no influence on the data collection, data assembly, data analysis, or the content of this paper.

## CONFLICT OF INTEREST STATEMENT

P.A.F. received personal fees from Novartis, Pfizer, Daiichi Sankyo, AstraZeneca, Eisai, Merck Sharp & Dohme, Lilly, SeaGen, Roche, Agendia, Gilead, Mylan, Menarini, Veracyte, GuardantHealth, and grants from Biontech, Pfizer, Cepheid, during the conduct of the study; and Translational Research in Oncology (TRIO). C.C.H. received honoraria from AstraZeneca, Daiichi Sankyo, Eisai, Novartis, Pfizer, Roche, Gilead, and MSD, and travel grants from Daiichi Sankyo. B.A. received honoraria from AstraZeneca, Gilead, Genomic Health, Roche, Novartis, Celgene, Lilly, MSD, Eisai, Stemline, Teva, Tesaro, Daiichi Sankyo, and Pfizer. S.K. reports personal fees from AstraZeneca, Pfizer, Lilly, Amgen, Hologic, Daiichi Sankyo, MSD Oncology, Sonoscape, Gilead Sciences, Agendia, Roche, Novartis, Exact Sciences, PINK, Celgene, and an uncompensated relationship with WSG. N.N. is currently an employee of AstraZeneca UK Limited and an employee of Novartis Pharma GmbH in the past. C.T. reports being a committee member of AGO, S3‐guideline breast cancer, ESMO, being a co‐editor in chief of BREAST CARE, and receiving lecture/travel fees for lectures at Essener Symposium zur Gynäk. Onkologie und Senologie, Essen, Germany, streamedup! GmbH, Wiesbaden, Germany, Rottalinnkliniken, Eggenfelden, Germany, Universitätsspital Basel, Basel, Switzerland, Onkowissen TV, ESMO congress, Deutsche Gesellschaft für Senologie e.V. H.‐C.K. received honoraria from Pfizer, Novartis, Roche, Genomic Health/Exact Sciences, Amgen, AstraZeneca, Riemser, Carl Zeiss Meditec, TEVA, Theraclion, Janssen‐Cilag, GSK, LIV Pharma, Lilly, Daiichi Sankyo, Gilead, and Zuellig, travel support from Carl Zeiss Meditec, LIV Pharma, Novartis, Amgen, Pfizer, Daiichi Sankyo, Tesaro, Gilead, AstraZeneca, Zuellig, and Stemline, participated in data safety monitoring or advisory boards for Pfizer, Novartis, SurgVision, Carl Zeiss Meditec, Amgen, Onkowissen, MSD, Gilead, Daiichi Sankyo, Seagen, Genomic Health/Exact Sciences, Agendia, Lilly, and owns stock of Theraclion SA. W.J. has received research grants and/or honoraria from AstraZeneca, Celgene, Chugai, Daiichi Sankyo, Eisai, Exact Sciences, Gilead, GSK, Guardant Health, Janssen, Lilly, Menarini, Stemline, MSD, NeoGenomics, Novartis, Pfizer, Roche, Sanofi‐Aventis, Seagen. A.S. received honoraria from Amgen, AstraZeneca, Aurikamed, Bayer, Celgene, ClinSol GmbH & Co. KG, Clovis Oncology, coma UroGyn, Connectmedica, Daiichi Sankyo, Gilead, GSK, if‐kongress, I‐MED, iOMEDICO, Lilly, MCI Deutschland, med publico, Metaplan, MSD, Mylan, NanoString Technologies, Novartis, onkowissen.de, Pfizer, Pierre Fabre, promedicis, Roche, Seagen, streamedup, SYNLAB, Tesaro, and travel support from AstraZeneca, Celgene, Daiichi Sankyo, Gilead, Pfizer, Roche. M.W.S. received honoraria from AstraZeneca, Pfizer, Clovis, Mylan, Roche, Gedeon Richter, Carl Zeiss Meditec, travel support from Pfizer, and Carl Zeiss Meditec. C.J. reports travel grants and honoraria from Roche, Novartis, Lilly, AstraZeneca, and Exact Sciences. V.M. received speaker honoraria from AstraZeneca, Daiichi Sankyo, Eisai, Pfizer, MSD, Medac, Novartis, Roche, Seagen, Onkowissen, high5 Oncology, Medscape, Gilead, and Pierre Fabre, iMED Institut. Consultancy honoraria: Roche, Pierre Fabre, PINK, ClinSol, Novartis, MSD, Daiichi‐Sankyo, Eisai, Lilly, Seagen, Gilead, Stemline. Institutional research support from Novartis, Roche, Seagen, Genentech, and AstraZeneca. Travel grants from AstraZeneca, Roche, Pfizer, Daiichi Sankyo, and Gilead. C.R. received honoraria from MSD and AstraZeneca, travel expenses from the Swiss Society of Senology and the Swiss Society of Gynecology. P.D. received honoraria from Novartis, MSD, Pierre Fabre, AstraZeneca, Lilly, Gilead Sciences, Daiichi Sankyo, Eisai, and Polytech. E.B. received honoraria from Novartis, Hexal, BMS, Lilly, Pfizer, Roche, MSD, Bayer, Ipsen, Bluebird, B. Braun, and onkowissen.de for consulting, clinical research management, or medical education activities. S.Y.B. has received honoraria from Roche Pharma, Novartis, Pfizer, AstraZeneca, and Teva. T.N.F. has received honoraria from Novartis, Roche, Pfizer, Daiichi Sankyo, and MSD. H.H. received lecture fees from Novartis Pharma GmbH, LEO Pharma GmbH, Atlanta GmbH, and Lilly Deutschland GmbH. C.G. received speaker honoraria from Novartis Pharma GmbH and ClinSol GmbH & Co. KG. K.A. reports personal fees from Roche Pharma AG, Pfizer Pharma GmbH, Seagen, and AstraZeneca. C.W. received honoraria from Lilly, Novartis, Sandoz, Hexal, and Gilead. A.R. received payment for lectures and advisory boards from Roche, Daiichi Sankyo, AstraZeneca, Pfizer, Novartis, Celgene, Exact Sciences, MSD, Pierre Fabre, Lilly, Seagen, Amgen, and GSK. B.R. received research grants from Novartis. All of the remaining authors have declared that they do not have any conflicts of interest.

## ETHICS STATEMENT

The PreFace Study (Evaluation of PREdictive FACtors Regarding the Effectivity of Aromatase Inhibitor Therapy, NCT01908556) was a prospective, open‐label phase IV clinical trial in patients with HRpos eBC.^17^ Ethics Committee Approval was obtained from the Medical Faculty of the Friedrich‐Alexander University Erlangen‐Nuremberg (approval number 25_2008) and all involved ethics committees for the respective study sites. All patients provided a written informed consent.

## Supporting information


Data S1

## Data Availability

The datasets used and/or analyzed during the current study are available from the corresponding author on reasonable request.

## References

[1] Cortazar P , Zhang L , Untch M , et al. Pathological complete response and long‐term clinical benefit in breast cancer: the CTNeoBC pooled analysis. Lancet. 2014;384(9938):164‐172. doi:10.1016/S0140-6736(13)62422-8 24529560

[2] von Minckwitz G , Untch M , Blohmer JU , et al. Definition and impact of pathologic complete response on prognosis after neoadjuvant chemotherapy in various intrinsic breast cancer subtypes. J Clin Oncol. 2012;30(15):1796‐1804. doi:10.1200/JCO.2011.38.8595 22508812

[3] Fasching PA , Heusinger K , Haeberle L , et al. Ki67, chemotherapy response, and prognosis in breast cancer patients receiving neoadjuvant treatment. BMC Cancer. 2011;11:486. doi:10.1186/1471-2407-11-486 22081974 PMC3262864

[4] Huang M , O'Shaughnessy J , Zhao J , et al. Evaluation of pathologic complete response as a surrogate for long‐term survival outcomes in triple‐negative breast cancer. J Natl Compr Canc Netw. 2020;18(8):1096‐1104. doi:10.6004/jnccn.2020.7550 32755985

[5] Huang M , O'Shaughnessy J , Zhao J , et al. Association of pathologic complete response with long‐term survival outcomes in triple‐negative breast cancer: a meta‐analysis. Cancer Res. 2020;80(24):5427‐5434. doi:10.1158/0008-5472.CAN-20-1792 32928917

[6] Torrisi R , Marrazzo E , Agostinetto E , et al. Neoadjuvant chemotherapy in hormone receptor‐positive/HER2‐negative early breast cancer: when, why and what? Crit Rev Oncol Hematol. 2021;160:103280. doi:10.1016/j.critrevonc.2021.103280 33667658

[7] Loibl S , Andre F , Bachelot T , et al. Early breast cancer: ESMO clinical practice guideline for diagnosis, treatment and follow‐up. Ann Oncol. 2024;35(2):159‐182. doi:10.1016/j.annonc.2023.11.016 38101773

[8] U.S. Department of Health and Human Services Food and Drug Administration (FDA) . Pathological Complete Response in Neoadjuvant Treatment of High‐Risk Early‐Stage Breast Cancer: Use as an Endpoint to Support Accelerated Approval Guidance for Industry. https://wwwfdagov/media/83507/download 2020 accessed August 28, 2022.

[9] Esserman LJ , DeMichele A . Accelerated approval for Pertuzumab in the neoadjuvant setting: winds of change? Clin Cancer Res. 2014;20(14):3632‐3636. doi:10.1158/1078-0432.Ccr-13-3131 24748554

[10] Conforti F , Pala L , Sala I , et al. Evaluation of pathological complete response as surrogate endpoint in neoadjuvant randomised clinical trials of early stage breast cancer: systematic review and meta‐analysis. BMJ. 2021;375:e066381. doi:10.1136/bmj-2021-066381 34933868 PMC8689398

[11] Karagiannis GS , Condeelis JS , Oktay MH . Chemotherapy‐induced metastasis: molecular mechanisms, clinical manifestations, therapeutic interventions. Cancer Res. 2019;79(18):4567‐4576. doi:10.1158/0008-5472.CAN-19-1147 31431464 PMC6744993

[12] Karagiannis GS , Condeelis JS , Oktay MH . Chemotherapy‐induced metastasis: mechanisms and translational opportunities. Clin Exp Metastasis. 2018;35(4):269‐284. doi:10.1007/s10585-017-9870-x 29307118 PMC6035114

[13] Karagiannis GS , Pastoriza JM , Wang Y , et al. Neoadjuvant chemotherapy induces breast cancer metastasis through a TMEM‐mediated mechanism. Sci Transl Med. 2017;9(397):eaan0026. doi:10.1126/scitranslmed.aan0026 28679654 PMC5592784

[14] Karagiannis GS , Condeelis JS , Oktay MH . Chemotherapy‐induced metastasis in breast cancer. Oncotarget. 2017;8(67):110733‐110734. doi:10.18632/oncotarget.22717 29340008 PMC5762276

[15] Early Breast Cancer Trialists' Collaborative Group . Long‐term outcomes for neoadjuvant versus adjuvant chemotherapy in early breast cancer: meta‐analysis of individual patient data from ten randomised trials. Lancet Oncol. 2018;19(1):27‐39. doi:10.1016/S1470-2045(17)30777-5 29242041 PMC5757427

[16] Gianni L , Baselga J , Eiermann W , et al. Phase III trial evaluating the addition of paclitaxel to doxorubicin followed by cyclophosphamide, methotrexate, and fluorouracil, as adjuvant or primary systemic therapy: European cooperative trial in operable breast cancer. J Clin Oncol. 2009;27(15):2474‐2481. doi:10.1200/JCO.2008.19.2567 19332727

[17] Hack CC , Maass N , Aktas B , et al. Long‐term follow‐up and safety of patients after an upfront therapy with letrozole for early breast cancer in routine clinical care—the PreFace study. Geburtshilfe Frauenheilkd. 2024;84(2):185‐195. doi:10.1055/a-2238-3153 38344045 PMC10853028

[18] Wolff AC , Hammond ME , Schwartz JN , et al. American Society of Clinical Oncology/College of American Pathologists guideline recommendations for human epidermal growth factor receptor 2 testing in breast cancer. J Clin Oncol. 2007;25(1):118‐145. doi:10.1200/JCO.2006.09.2775 17159189

[19] Hammond ME , Hayes DF , Dowsett M , et al. American Society of Clinical Oncology/college of American pathologists guideline recommendations for immunohistochemical testing of estrogen and progesterone receptors in breast cancer. J Clin Oncol. 2010;28(16):2784‐2795. doi:10.1200/JCO.2009.25.6529 20404251 PMC2881855

[20] Gianni L , Pienkowski T , Im YH , et al. 5‐year analysis of neoadjuvant pertuzumab and trastuzumab in patients with locally advanced, inflammatory, or early‐stage HER2‐positive breast cancer (NeoSphere): a multicentre, open‐label, phase 2 randomised trial. Lancet Oncol. 2016;17(6):791‐800. doi:10.1016/S1470-2045(16)00163-7 27179402

[21] Korde LA , Somerfield MR , Carey LA , et al. Neoadjuvant chemotherapy, endocrine therapy, and targeted therapy for breast cancer: ASCO guideline. J Clin Oncol. 2021;39(13):1485‐1505. doi:10.1200/JCO.20.03399 33507815 PMC8274745

[22] Marme F , Lederer B , Blohmer JU , et al. Utility of the CPS+EG staging system in hormone receptor‐positive, human epidermal growth factor receptor 2‐negative breast cancer treated with neoadjuvant chemotherapy. Eur J Cancer. 2016;53:65‐74. doi:10.1016/j.ejca.2015.09.022 26693900

[23] Michel LL , Sommer L , Gonzalez Silos R , et al. Locoregional risk assessment after neoadjuvant chemotherapy in patients with primary breast cancer: clinical utility of the CPS + EG score. Breast Cancer Res Treat. 2019;177(2):437‐446. doi:10.1007/s10549-019-05314-9 31236813

[24] Roussot N , Constantin G , Desmoulins I , et al. Prognostic stratification ability of the CPS+EG scoring system in HER2‐low and HER2‐zero early breast cancer treated with neoadjuvant chemotherapy. Eur J Cancer. 2024;202:114037. doi:10.1016/j.ejca.2024.114037 38554542

[25] Cardoso F , O'Shaughnessy J , McArthur H , et al. Abstract GS01‐02: phase 3 study of neoadjuvant pembrolizumab or placebo plus chemotherapy, followed by adjuvant pembrolizumab or placebo plus endocrine therapy for early‐stage high‐risk ER+/HER2− breast cancer: Keynote 756. Cancer Res. 2024;84(9 Supplement):GS01‐02.

[26] Rohan TE , Xue X , Lin HM , et al. Tumor microenvironment of metastasis and risk of distant metastasis of breast cancer. J Natl Cancer Inst. 2014;106(8):dju136. doi:10.1093/jnci/dju136 24895374 PMC4133559

[27] Sparano JA , Gray R , Oktay MH , et al. A metastasis biomarker (MetaSite breast score) is associated with distant recurrence in hormone receptor‐positive, HER2‐negative early‐stage breast cancer. NPJ Breast Cancer. 2017;3:42. doi:10.1038/s41523-017-0043-5 29138761 PMC5678158

[28] Chabner BA . Does chemotherapy induce metastases? Oncologist. 2018;23(3):273‐274. doi:10.1634/theoncologist.2017-0648 29523674 PMC5905696

[29] DeMichele A , Yee D , Esserman L . Mechanisms of resistance to neoadjuvant chemotherapy in breast cancer. N Engl J Med. 2017;377(23):2287‐2289. doi:10.1056/NEJMcibr1711545 29211674

[30] Early Breast Cancer Trialists' Collaborative Group . Comparisons between different polychemotherapy regimens for early breast cancer: meta‐analyses of long‐term outcome among 100,000 women in 123 randomised trials. Lancet. 2012;379(9814):432‐444. doi:10.1016/S0140-6736(11)61625-5 22152853 PMC3273723

[31] Hosseini‐Mellner S , Wickberg A , Karakatsanis A , Valachis A . Impact of neoadjuvant compared to adjuvant chemotherapy on prognosis in patients with hormone‐receptor positive / HER2‐negative breast cancer: a propensity score matching population‐based study. Breast. 2024;76:103741. doi:10.1016/j.breast.2024.103741 38759576 PMC11127261

