# Supplementary material for: Invasive disease‐free and overall survival after (neo)adjuvant chemotherapy in postmenopausal patients with hormone receptor‐positive, HER2‐negative early breast cancer treated with upfront letrozole: Experiences from the phase IV PreFace trial
Source: Int J Cancer. 2025 Jul 11;157(11):2363–73. doi: 10.1002/ijc.70037 (PMC12496005; doi:10.1002/ijc.70037)
Supplement: Supplementary file 1 — Data S1 [file IJC-157-2363-s001.pdf]

Supplementary Information

Invasive disease-free and overall survival after (neo)adjuvant chemotherapy in postmenopausal patients with hormone receptor-positive, HER2-negative early breast cancer treated with upfront letrozole – experiences from the Phase IV PreFace trial

Milena Beierlein, Lothar Häberle, Naiba Nabieva, Nicolai Maass, Bahriye Aktas, Sherko Kümmel, Christoph Thomssen, Christopher Wolf, Hans-Christian Kolberg, Cosima Brucker, Wolfgang Janni, Peter Dall, Andreas Schneeweiss, Frederik Marme, Marc W. Sütterlin, Matthias Ruebner, Anna-Katharin Theuser, Nadine M. Hofmann, Sybille Böhm, Katrin Almstedt, Sara Kellner, Paul Gass, Hans-Joachim Lück, Alexander Hein, Sabine Schmatloch, Matthias Kalder, Christoph Uleer, Ingolf Jurhasz-Böss, Volker Hanf, Christian Jackisch, Volkmar Müller, Brigitte Rack, Erik Belleville, Diethelm Wallwiener, Achim Rody, Claudia Rauh, Chistian M. Bayer, Sabrina Uhrig, Hanna Huebner, Chloë Goossens, Sara Y. Brucker, Carolin C. Hack, Tanja N. Fehm, Peter A. Fasching

Table of contents

Supplementary Tables ……………………………………………………………………2

Supplementary Table 1: Invasive disease-free survival (iDFS) and overall survival (OS) rates for patients who were treated with taxane……………………………………………….........2

Supplementary Table 2: Hazard ratios for invasive disease-free survival (iDFS) and overall survival (OS) comparing taxane-treated patients with adjuvant chemotherapy and taxane-treated patients with neoadjuvant chemotherapy……………………………………………..…3

Supplementary Table 3: Invasive disease-free survival (iDFS) and overall survival (OS) rates..4

Supplementary Figures……………………………………………………………………5

Supplementary Figure 1: Survival of hormone receptor-positive / HER2-negative early breast cancer patients that received taxane-based neoadjuvant chemotherapy or taxane-based adjuvant chemotherapy A. Invasive disease-free survival. B. Overall survival………………..5

Supplementary Figure 2: Survival of hormone receptor-positive / HER2-negative early breast cancer patients based on receiving neoadjuvant chemotherapy, adjuvant chemotherapy or no chemotherapy. A. Invasive disease-free survival. B. Overall survival…………………….……..6

Supplementary Tables

Supplementary Table 1: Invasive disease-free survival (iDFS) and overall survival (OS) rates for patients who were treated with taxane.

Target	Prior chemotherapy	Patients	Events	2-year survival rate (95% CI)	3-year survival rate (95% CI)	5-year survival rate (95% CI)
iDFS	Adjuvant	593	72	0.96 (0.94, 0.97)	0.93 (0.91, 0.95)	0.87 (0.84, 0.90)
iDFS	Neoadjuvant	160	28	0.90 (0.85, 0.95)	0.85 (0.80, 0.91)	0.81 (0.75, 0.88)
OS	Adjuvant	593	44	0.98 (0.96, 0.99)	0.97 (0.95, 0.98)	0.92 (0.90, 0.94)
OS	Neoadjuvant	160	16	0.95 (0.92, 0.99)	0.91 (0.87, 0.96)	0.90 (0.85, 0.95)

CI confidence interval

Supplementary Table 2: Hazard ratios for invasive disease-free survival (iDFS) and overall survival (OS) comparing taxane-treated patients with adjuvant chemotherapy and taxane-treated patients with neoadjuvant chemotherapy.

Target	Prior chemotherapy	Hazard ratio adjusted[1] (95% CI)	Hazard ratio unadjusted (95% CI)
iDFS	Adjuvant	Reference	Reference
iDFS	Neoadjuvant	1.81 (1.15, 2.86)	1.47 (0.95, 2.28)
OS	Adjuvant	Reference	Reference
OS	Neoadjuvant	1.61 (0.88, 2.93)	1.35 (0.76, 2.40)

CI confidence interval

[1]Hazard ratio is adjusted for age, body mass index, lymph node status, grading, tumor size and histology.

Supplementary Table 3: Invasive disease-free survival (iDFS) and overall survival (OS) rates.

Target	Prior chemotherapy	Patients	Events	2-year survival rate (95% CI)	3-year survival rate (95% CI)	5-year survival rate (95% CI)
iDFS	Adjuvant	874	96	0.96 (0.94, 0.97)	0.94 (0.92, 0.95)	0.88 (0.85, 0.90)
iDFS	Neoadjuvant	177	30	0.89 (0.85, 0.94)	0.85 (0.80, 0.91)	0.81 (0.75, 0.88)
iDFS	Naive	1,844	161	0.97 (0.96, 0.97)	0.95 (0.94, 0.96)	0.91 (0.89, 0.92)
OS	Adjuvant	874	53	0.98 (0.97, 0.99)	0.97 (0.96, 0.99)	0.93 (0.91, 0.95)
OS	Neoadjuvant	177	18	0.95 (0.92, 0.98)	0.91 (0.87, 0.96)	0.89 (0.85, 0.94)
OS	Naive	1,844	54	0.99 (0.99, 1.00)	0.99 (0.98, 0.99)	0.97 (0.96, 0.98)

CI confidence interval

Supplementary Figures

A. Invasive disease-free survival relative to type of chemotherapy

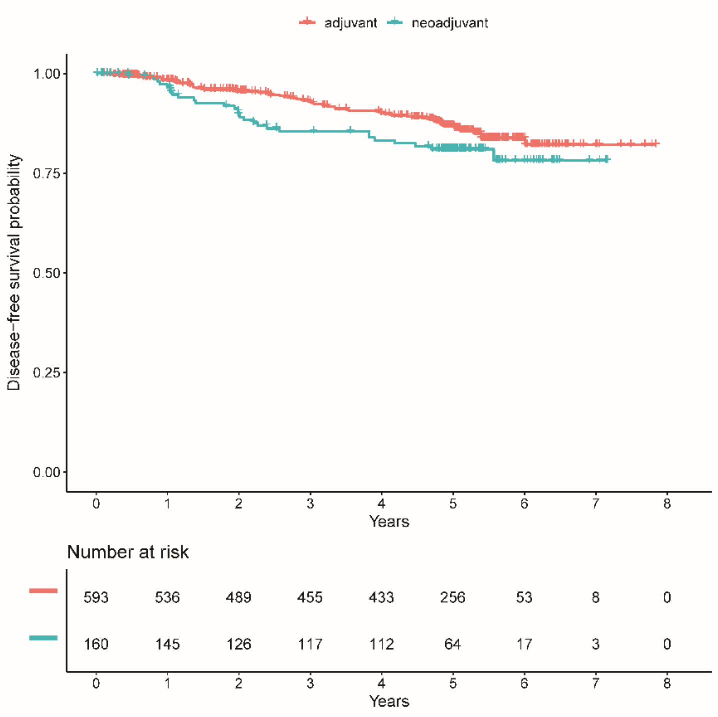

B. Overall survival relative to type of chemotherapy

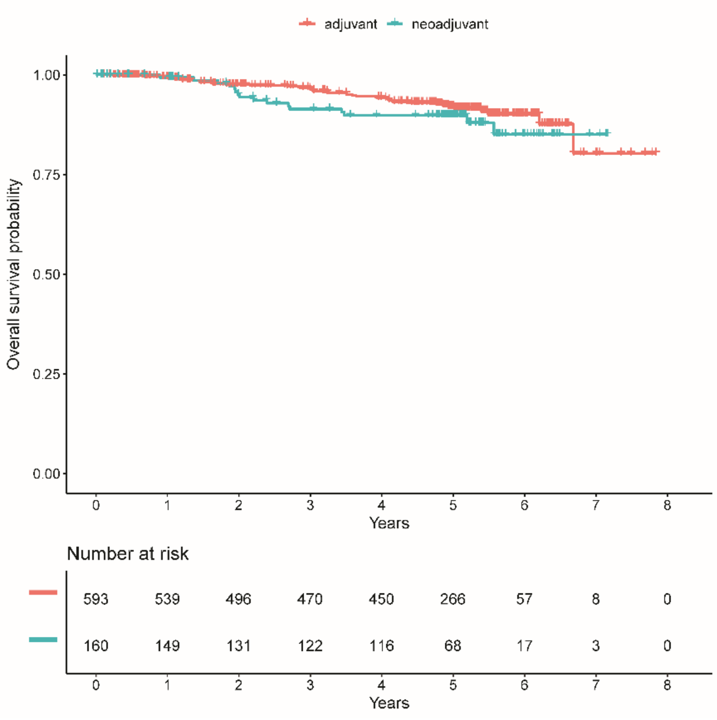

Supplementary Figure 1. Survival of hormone receptor-positive / HER2-negative early breast cancer patients that received taxane-based neoadjuvant chemotherapy or taxane-based adjuvant chemotherapy A. Invasive disease-free survival. B. Overall survival.

A. Invasive disease-free survival relative to type of chemotherapy

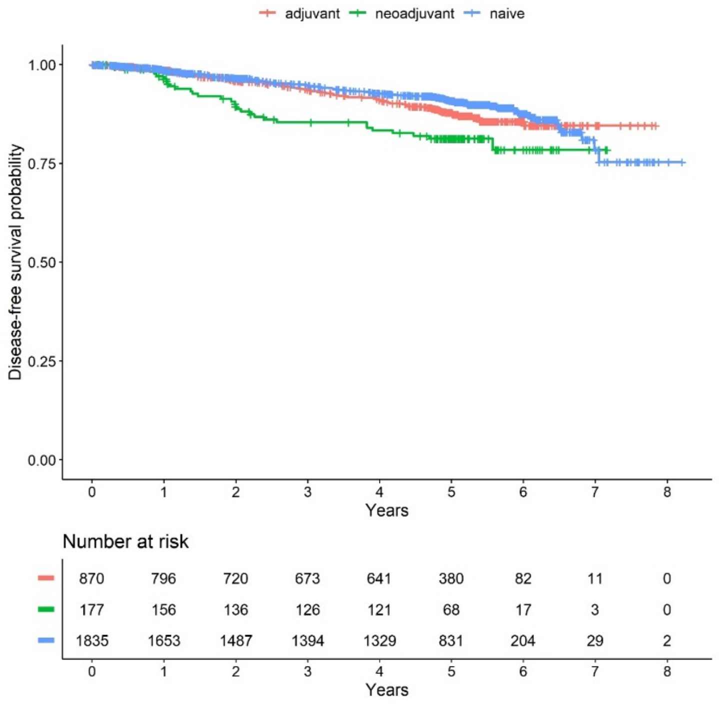

B. Overall survival relative to type of chemotherapy

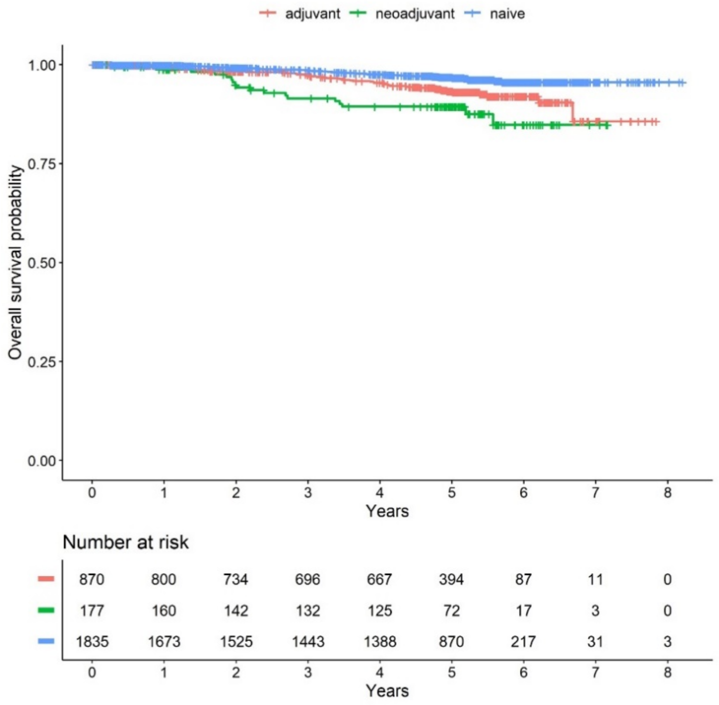

Supplementary Figure 2. Survival of hormone receptor-positive / HER2-negative early breast cancer patients based on receiving neoadjuvant chemotherapy, adjuvant chemotherapy or no chemotherapy. A. Invasive disease-free survival. B. Overall survival.
